# Synthetic probes for the study of biological function

**DOI:** 10.3762/bjoc.9.10

**Published:** 2013-01-15

**Authors:** Jeffrey Aubé

**Affiliations:** 1 Department of Medicinal Chemistry, the University of Kansas, 2034 Becker Drive, Lawrence, Kansas, 66047, USA

From its beginnings, organic chemistry has been a partner to biology, often crossing the artificial boundary between the sciences to the benefit of both disciplines. This is even reflected in the use of the adjective “organic” to refer to the chemistry of carbon. As the field has matured, it has continued to address matters of *structure* and *synthesis* to increasingly encompass the grand challenge of designing and preparing molecules having a particular *function*. All three of these come together in the pursuit of new chemical probes for use in chemical biology.

This Thematic Series highlights some of the numerous ways that molecular tools inform biological research. The choice of the phrase “chemical probe” was deliberately vague and indeed its meaning often lies in the ears of the listener. The most common context is the case of a molecule used in chemical biology to help unravel the role of a particular cellular pathway. Biologists have numerous tools at their disposal, of course, and one important reason to select a small organic molecule as an inhibitor, say, is that it allows for temporal control of its effect. Of course, inhibitors (and other sorts of biologically active molecules) have a way of occasionally – very occasionally – turning into drugs, and this offers its own rationale for research in this direction. Regardless of the goal, the challenge of finding a small molecule that binds to a macromolecular target in a specific enough way to influence its function is considerable.

The combination of a binding event with a physical property such as fluorescence leads to another sort of probe, where the goal is to inform the researcher where a particular target may exist in a cell. In addition, the advent of phenotypic screening has created a strong demand for probes that can be used to help identify the target at which a screening hit is working. Each provides challenges to the chemist as it is necessary to retain the key binding event in such a way that also permits a useful read-out or physical capture of the intended target.

Meeting challenges such as those described above requires both biological insight and chemical know-how. It has often been said that a distinguishing characteristic of chemistry is its ability to introduce new forms of matter into the universe, and we now have over a hundred years of accumulated experience in doing just that. The structural diversity of molecules that function as probes is broad indeed, some of them complex enough that even the most discerning practitioner of organic synthesis may consider them as worthy of their efforts.

I would like to extend my most sincere thanks to the authors and reviewers who have made this Thematic Series possible and for their support of open access publication in organic chemistry. I hope that this Thematic Series will inspire many of its readers to lend their talents to this still-emerging branch of *chemistry*.

Jeff Aubé

Lawrence, January 2013

